# Mesenchymal stem cells support human vascular endothelial cells to form vascular sprouts in human platelet lysate-based matrices

**DOI:** 10.1371/journal.pone.0278895

**Published:** 2022-12-15

**Authors:** Sabrina Summer, Eva Rossmanith, Markus Pasztorek, Constantin Fiedler, Marion Gröger, Sabine Rauscher, Viktoria Weber, Michael B. Fischer

**Affiliations:** 1 Department for Biomedical Research, Center of Experimental Medicine, University for Continuing Education Krems, Krems an der Donau, Austria; 2 Clinic for Blood Group Serology and Transfusion Medicine, Medical University of Vienna, Vienna, Austria; 3 Core Facility Imaging, Medical University of Vienna, Vienna, Austria; 4 Department for Biomedical Research, Center for Biomedical Technology, University for Continuing Education Krems, Krems an der Donau, Austria; Affiliated Hospital of Jiangsu University, CHINA

## Abstract

During tissue regeneration, mesenchymal stem cells can support endothelial cells in the process of new vessel formation. For a functional interaction of endothelial cells with mesenchymal stem cells a vascular inductive microenvironment is required. Using a cellular model for neo-vessel formation, we could show that newly formed vascular structures emanated from the embedded aggregates, consisting of mesenchymal stem cells co-cultured with autologous human umbilical vein endothelial cells, into avascular human platelet lysate-based matrices, bridging distances up to 5 mm to join with adjacent aggregates with the same morphology forming an interconnected network. These newly formed vascular sprouts showed branch points and generated a lumen, as sign of mature vascular development. In two-dimensional culture, we detected binding of mesenchymal stem cells to laser-damaged endothelial cells under flow conditions, mimicking the dynamics in blood vessels. In conclusion, we observed that mesenchymal stem cells can support human umbilical vein endothelial cells in their vitality and functionality. In xeno-free human platelet lysate-based matrices, endothelial cells form complex vascular networks in a primarily avascular scaffold with the aid of mesenchymal stem cells, when co-cultured in three-dimensional spherical aggregates. Under dynamic conditions, representing the flow rate of venous vessel, mesenchymal stem cells preferably bind to damaged endothelial cells presumably assisting in the healing process.

## Introduction

Mesenchymal stem cells (MSCs) are the stem cells of the connective tissue, showing a strong regenerative activity to counteract degenerative diseases due to their multi-lineage differentiation and self-renewal capability [1–4]. MSCs support the regeneration of the connective tissue by integrating into the damaged site and maturing along the mesenchymal lineage towards organ-specific cells. MSCs can release their secretome or emit microvesicles (MVs) and exosomes into damaged tissues, or donate mitochondria to rescue damaged cells [5, 6]. Finally, MSCs contribute to tissue and organ regeneration by supporting endothelial cells (ECs) in the process of new vessel formation [7–12]. Sprouting angiogenesis in response to ischemia and hypoxia starts with extracellular matrix (ECM) degradation and detachment of perivascular MSCs from capillaries in the affected area. This allows the endothelial tip cell to become invasive and induces the formation of filopodia and lamellipodia in response to guidance cues determining the direction of vascular growth [13–15]. After initiation, stalk cells, that lie behind the tip cells, tend to proliferate, and extend the vessel by forming the ECM, junctions, and a lumen [14, 15]. Once the tip cells anastomose with other tip cells to form a potential circuit, blood vessel maturation takes place, which involves the recruitment of endothelial precursors from the circulation [14]. This favors deposition of ECM by ECs and perivascular MSCs, and the initiation of a blood flow [13]. Under these conditions, perivascular MSCs come into direct contact with immature capillaries to stabilize the newly formed tubular network by abluminal coverage [15]. Vascular basement membranes can directly interact with ECs that line the inside, and perivascular MSCs covering the outside of the newly formed blood vessel [15]. MSCs have been shown to produce and deposit collagen type IV, a major constituent of vascular basement membranes, within newly formed blood vessels *in vivo* and in fibrin-based matrices [16, 17]. During the integration into the vascular wall, MSCs respond to EC-derived TGF-β [18, 19]. Contact-dependent activation of TGF-β is a common driver of MSC differentiation into vascular smooth muscle cells during vessel maturation, involving the TGF-β/ALK signaling pathway [19].

Important for the MSC-mediated support of ECs in angiogenesis models are the tissue origin of the MSCs, their cultivation conditions and the scaffold composition [1–4]. Among different EC types investigated, human umbilical vein endothelial cells (HUVECs) were extensively studied in co-culture with MSCs, smooth muscle cells, or pericytes as supportive cells [7–12]. While bone marrow-derived and adipose-derived MSCs supported HUVEC organization into vascular-like structures, amniotic fluid-derived MSCs appeared to provide enhanced pro-vascular support, indicating the importance of an earlier stage in ontogeny [8, 12]. Cultivation of MSCs in adherence to plastic surface combined with xeno-based medium supplements could substantially modify the phenotype and transcriptional activity of the cells, resulting in an impaired interaction with HUVECs in angiogenesis models [20]. In search of xeno-free cultivation solutions, human platelet lysate (HPL)-based medium supplements were used to maintain the phenotype of cultured MSCs, reduce stress fiber formation and preserve mitochondrial function [21]. However, current knowledge assessing the effects of HPL-based matrices on the communication between ECs and MSCs during vessel formation is limited.

Here, we investigated the potential of amnion-derived MSCs to support HUVECs in the process of *de novo* vessel formation using primary avascular HPL-based matrices, providing a xeno-free system in an autologous setting using cells isolated from the same donor material. HPL derived from platelets of healthy human donors is rich in growth factors, such as platelet-derived growth factors, brain-derived neurotrophic factor and epidermal growth factor [21], and can be used as an alternative to xeno-based growth medium supplements for cell propagation [22, 23]. HPL-based scaffolds combine the characteristics of fibrin-based matrices with the bioactive content of platelets, including factors mediating angiogenesis and supporting cell-cell or cell-matrix interactions [21].

Spherical aggregates, containing a defined ratio of autologous MSCs and HUVECs and generated by the hanging drop technology, were embedded into a gel containing 20% HPL to investigate potential vessel formation. As developmental signs of vascular maturation, the capability of these newly formed vascular structures to generate tube-like structures and bridge distances of up to 5 mm to the neighboring spheroid with the same morphology was observed.

Further, we investigated the dynamic adhesion of MSCs to damaged HUVECs in a microfluidic system applying a flow rate representing the blood flow in venous vessels. The MSCs preferably bind to the damaged HUVECs, presumably aiding in their regeneration.

## Materials and methods

### Isolation, cultivation, and characterization of MSCs and HUVECs

Human placental tissues were obtained from healthy delivering women in accordance with the Austrian Hospital Act (KAG 1982) after written informed consent and the study was approved by the Ethic Commission of Lower Austria (GSl-EK-4/3122015). Amnion-derived MSCs from placental tissue were isolated and characterized with CD73-APC, CD90-FITC and CD105-PE-Cy7 (all from eBioscience, San Diego, CA) by flow cytometry (CytoFLEX XL, Beckman Coulter GmbH, Krefeld, Germany) as described previously [21] and HUVECs were isolated from the umbilical vein and characterized as published previously [24].

### Generation of spherical aggregates in hanging drops

Spherical aggregates were generated by co-cultivation of 4500 MSCs and 500 HUVECs (isolated from the same donor material) on lids of petri dishes (Greiner Bio-One, Kremsmünster, Austria) using the hanging drops technology [21]. The cell number was determined using the Luna Automated Cell Counter (Logos Biosystems, South Korea). Cells were co-cultivated in a volume of 25 μI M-199 medium supplemented with 10% fetal bovine serum (Gibco, Thermo Fisher Scientific, USA), endothelial growth supplement (20 μg/ml, Becton Dickinson, USA) and heparin (10 IU/ml, Baxalta, Austria). Alternatively, MSC aggregates of 5000 MSCs were cultured in MSC-BM^TM^ or MSC-GM^TM^ (both from Lonza Group Ltd, Switzerland) using the same technology and monitoring of aggregate formation was performed by phase contrast microscopy (IMT2, Olympus Austria GmbH, Austria) equipped with a digital camera (DP50, Olympus). A detailed protocol is provided here dx.doi.org/10.17504/protocols.io.bp2l6138kvqe/v1.

### Imaging of spherical aggregates by scanning electron microscopy

MSC aggregates were adhered on Nunc Thermanox^TM^ coverslips (Nunc, Thermo Fisher Scientific, USA), fixed, dehydrated, mounted on conductive double side adhesive carbon tabs (Miere to Nano V.O.F., Netherlands), sputtered with gold and analyzed by a scanning electron microscope (FlexSEM, Hitachi Ltd. Corp., Japan) as previously described [21].

### Imaging of spherical aggregates by confocal microscopy

MSC aggregates were cultivated in Nunc^TM^ Lab-Tek^TM^ II chamber slides (Nunc, Thermo Fisher Scientific, USA), fixed and permeabilized after 16 hours and stained with Alexa Fluor AF^®^ 594 phalloidin 1:200 (0.1 U/ml, Molecular Probes, Thermo Fisher Scientific, USA) to reveal filamentous (f)-actin. To stain focal adhesions, a mouse mAb to paxillin and a mouse mAb vinculin both 1:100 (both from Santa Cruz Biotechnology, USA) followed by goat-anti-mouse Fab fragments labeled with AF^®^ 488 1:500 (3 μg/ml, Jackson Laboratories, USA) were used, and nuclei were stained with DAPI 1:1000 (Sigma-Aldrich, USA). As endothelial marker von Willebrand factor (vWF) mAb (2 μg/ml, Dianova, Germany) 1:100, followed by goat-anti-rabbit Fab fragments labeled with AF^®^ 488 1:500 (3 μg/ml, Jackson Laboratories, USA) were used. The slides were mounted with Fluoromount-G^TM^ (Southern Biotechnology, Thermo Fisher Scientific, USA) and analyzed with an Apochromat 63x objective on a confocal microscope (TCS SP8, Leica Microsystem GmbH, Germany) using the LASX-software version 3.1.5–16308 [21].

### Extracellular matrix composition of spherical aggregates by confocal microscopy

MSC aggregates were embedded in optimal cutting temperature (OCT) compound (Tissue Tek, Sakura Finetek Europe BV, Netherlands) and shock frozen in liquid nitrogen to generate cryosections of 4 μm on a Cryostar NX70 (Thermo Fisher Scientific, USA). Sections were mounted on Superfrost Plus slides (Menzel Glas, VWR, Thermo Fisher Scientific, USA), fixed in 4°C-precooled acetone and stored at -80°C. ECM components were detected using mouse mAbs specific for collagen type I (0.5 μg/ml clone ab90395, Abcam^®^, UK), collagen type IV (2 μg/ml clone M0785, Dako, Agilent Pathology Solutions, USA), fibronectin (2 μg/ml clone MAB1918, R&D Systems, USA), or a rabbit mAb specific for laminin (5 μg/ml clone ab11575, Abcam^®^, UK). Primary mAbs were subsequently stained either with goat anti-mouse lgG AF^®^ 594 or with goat anti-rabbit lgG AF^®^ 594 (both 1:500, Jackson Laboratories, USA). MSCs were counter-stained with CD90-FITC 1:100 (eBioscience, USA) and nuclei were stained with DAPI 1:1000 (Sigma-Aldrich, USA). The slides were mounted with Fluoromount-G^TM^ (Southern Biotechnology, USA) and analyzed on a confocal microscope (SP8, Leica, Germany) [21].

### Vascular network formation in HPL-based matrices by confocal microscopy

Human thrombin (20 U/ml, Sigma-Aldrich, Germany) was added to 20% human platelet lysate (HPL, MacoPharma, France) in M-199 Medium supplemented with endothelial cell growth supplement (ECGS) and 500 μl of the mix was pipetted onto an ibidi μ-Dish (ibidi GmbH, Germany). After gel formation occurred, spherical aggregates consisting of MSCs and HUVECs were embedded on the gel surface at positions with 2–5 mm between the aggregates (Fig 3 scale bar). After 48 hours 500 μl of M-199 medium supplemented with 8% HPL and ECGS was added, and medium was exchanged once a week. New vessel formation was monitored twice a week using the ChemiDoc system (Bio-Rad Laboratories Inc., USA) and phase contrast microscopy (IMT-2, Olympus). After 21 days of cultivation, HPL-based gels were fixed with 4% formaldehyde overnight to guarantee gel integrity. Cells within the HPL-based gels were stained with AF^®^ 488 phalloidin (0.1 U/ml, Molecular Probes, USA) and HUVECs with a rabbit anti-human von Willebrand factor (vWF) mAb (1:100, 2 μg/ml, Dianova, Germany) followed by goat anti-rabbit lgG Alexa Fluor^®^ 594 (1:500, Jackson Laboratories, USA). Nuclei were stained with DAPI 1:1000 (Sigma-Aldrich, USA). Alternatively, before spheroid formation by hanging drops technique, MSCs were labeled with Cell Tracker^TM^ Green CMFDA dye (1:1000 dilution) and HUVECs were labeled with Cell Tracker^TM^ Red CMTPX dye (1:1000 dilution, both from Thermo Fisher Scientific, USA). The gels were covered with 500 μl PBS and z-stack analysis was performed using an Apochromat 10x objective and confocal microscopy (SP8, Leica, Germany).

### Binding of MSCs to adherent HUVECs in flow cells of the BioFlux^®^200 device

An electro pneumatically controlled BioFlux^®^200 system (Fluxion Biosciences, lnc., USA) with microfluidic plates of 24 independent flow chambers with a dimension of 350 μm width, 1500 μm length and 70 μm height was used to investigate binding of MSCs to a HUVEC layer applying 2 dyne/cm^2^ flow. Flow channels were coated with fibronectin (2.5 μg/cm^2^, Gibco, Thermo Fisher Scientific, USA) and 2*10^5^ HUVECs in a volume of 200 μl EGM-2 medium (Lonza Group Ltd, Switzerland) were seeded and cultivated overnight under static conditions. To reach confluency of the HUVEC layer, perfusion with 2 dyne/cm^2^ was applied for 24 to 48 hours. Damage to the HUVEC layer in an area of 150 μm in diameter (0.0176 mm^2^) was introduced by a laser capture micro-dissector (LMD6, Leica Microsystems GmbH, Germany). After dead HUVECs were removed, MSCs were applied via the inlet terminal in a density of 1*10^5^ cells/ml under 2 dyne/mm^2^ flow rate. Binding of MSCs to the HUVEC layer was monitored by phase contrast microscopy and the reaction was stopped after 3 hours by fixation with 4% formaldehyde. Flow cells were washed and incubated with mouse anti-human VE-cadherin (2 μg/ml clone 123413, R&D Systems, US) followed by goat anti-mouse lgG AF^®^ 594 (1:500, Jackson Laboratories, USA) to stain tight junctions of ECs and CD90 FITC (2.5 μg/ml, eBioscience, USA) to stain MSCs. Nuclei were stained with DAPI. Binding of MSCs to the EC layer was investigated using an LSM 700LS confocal laser scanning microscope (Carl Zeiss Microscopy GmbH, Germany) and the ZEN software program (Zeiss, Germany). A reference area of 0.1 mm^2^ (elliptic area of 330 x 415.5 μm) was investigated by phase contrast microscopy to quantify MSC binding to the HUVEC layer.

### Statistical analysis

The statistical analysis was performed in GraphPad Prism 7.02. Details on the sample size and performed statistical analysis are specified in the corresponding figure legends. Statistical significance is indicated in the figure * p<0.05.

## Results

### MSCs generate delicate actin filaments within 3D spherical aggregates and contribute to the extracellular matrix for stability

**Fig 1:**
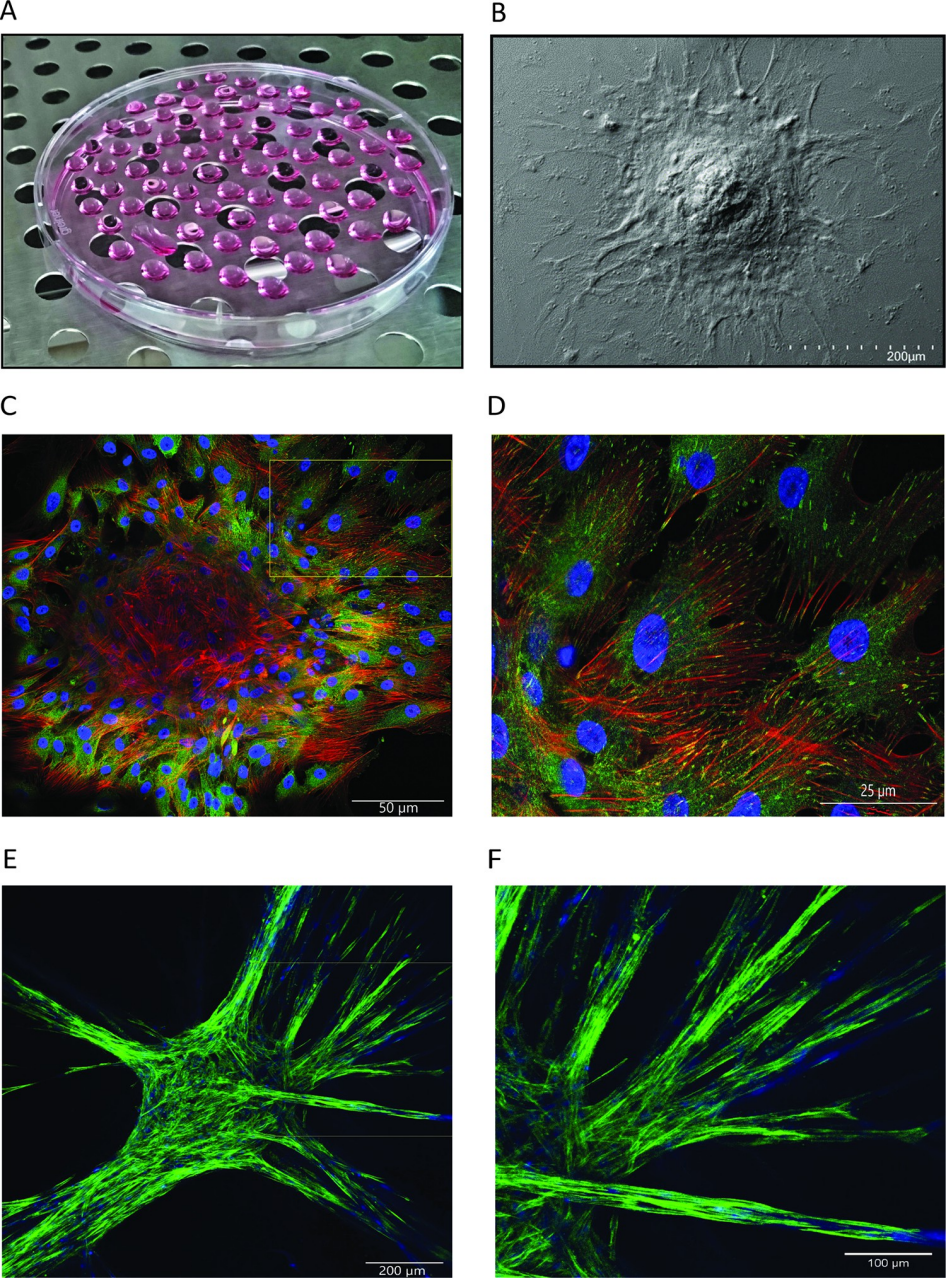
Spherical aggregate of MSCs. Spherical aggregates generated by cultivating 5000 MSCs on the lid of a petri dish using the hanging drop technology (A). Representative image of a spherical MSC aggregate adherent to a coverslip obtained by scanning electron microscopy (B) and image of a spherical MSC aggregate stained with a mAb specific for paxillin to reveal focal adhesions (green), with phalloidin to stain actin filaments (red), and DAPI to highlight the nuclei (blue) generated by laser scanning microscopy (C, D). Image of a spherical MSC aggregate embedded in HPL matrices showing sprout formation after 14 days of cultivation. The actin filaments were stained with phalloidin (green) and nuclei with DAPI (blue) (E, F).

MSCs of passage 1, expressing the mesenchymal specific markers CD73, CD90 and CD105, were used to generate spherical aggregates applying the hanging drops technology (Fig 1A and S1 Fig). We showed that 5000 MSCs form stable 3D spherical aggregates using scanning electron microscopy and confocal microscopy (Fig 1B and 1C). When spherical MSC aggregates were adhered to plastic surface for imaging, MSCs emanated from the aggregates and adhered as single cells (Fig 1D). Those single MSCs developed ventral stress fibers that were anchored at both sides to focal adhesions, spanning the entire cell and showed actin branching as described previously (Fig 1E) [21].

**Fig 2:**
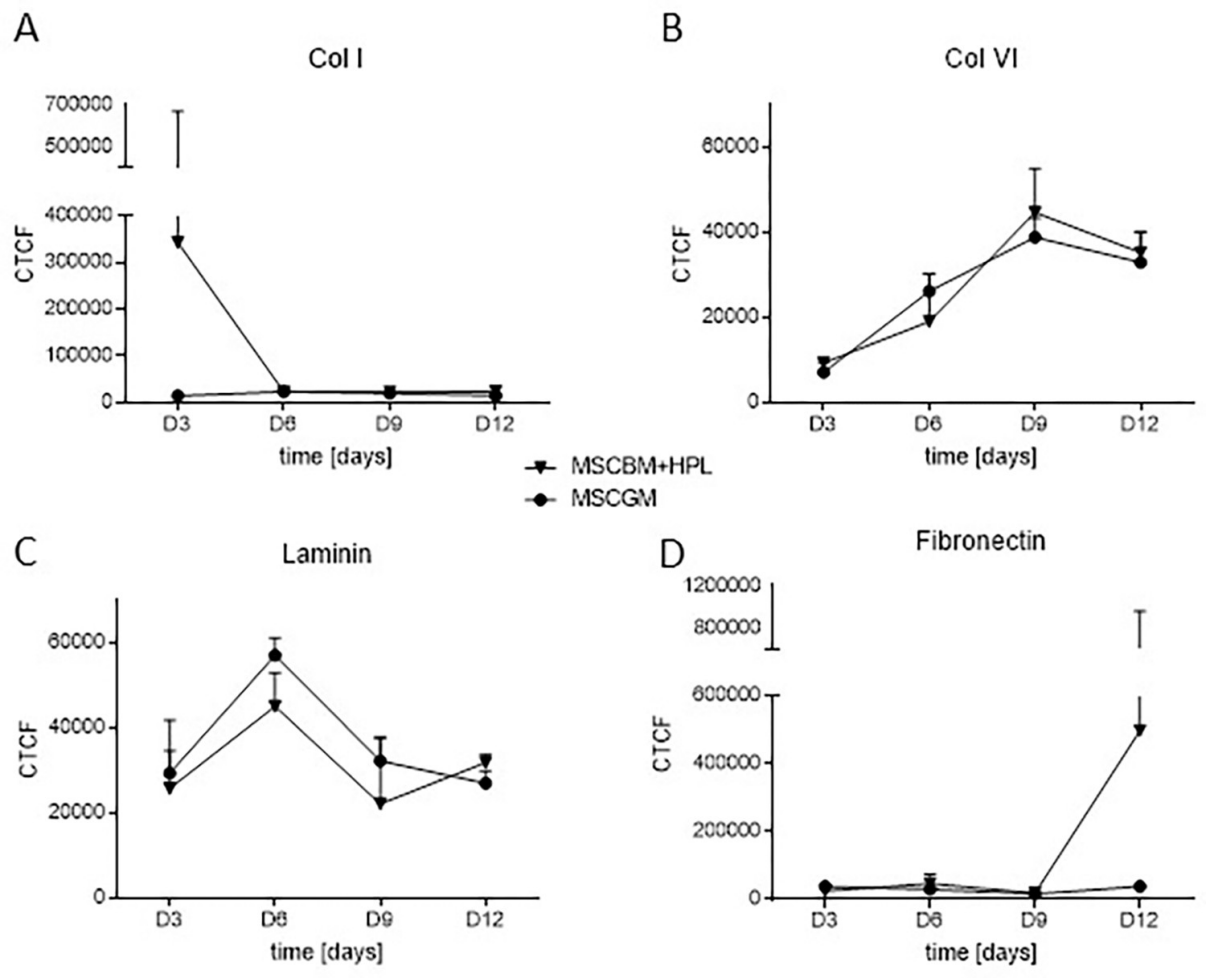
Calculation of extracellular matrix components in spherical aggregates cultivated in MSC-BM with 8% HPL and MSC-GM. The fluorescence intensity of collagen type I (A), collagen type IV (B), laminin (C) and fibronectin (D) were measured in aggregates grown in MSC-BM+HPL and MSC-GM for 3, 6, 9 and 12 days based on confocal microscopy images using ImageJ. To compare the expression of the ECM components in aggregates grown in MSC-BM+HPL to MSC-GM a two-way ANOVA (n = 2 for each day) was performed using GraphPad Prism 7. * *p*<0.05.

When spherical MSC aggregates were cultured in xeno-based medium, such as MSC-GM, MSCs generated collagen type I, collagen type IV, laminin, and fibronectin as major ECM components for aggregate stability (S2 Fig). The amount of individual ECM components (collagen type I, collagen type IV, laminin, and fibronectin) produced by MSCs within the 3D spherical aggregates did not change substantially during the period of 21 days of cultivation, except for Col IV (Fig 2, Col I *p* = 0.47, Col IV *p* = 0.02, laminin *p* = 0.12, fibronectin *p* = 0.4104). When HPL was used as xeno-free medium supplement (MSC-BM+HPL, Fig 2 and S3 Fig) to culture MSC aggregates, the production of Col I, Col IV, laminin, and fibronectin by MSCs did not differ from MSCs cultivated in xeno-based medium (MSC-GM) (Fig 2 and S2 Fig, Col I *p* = 0.42, Col IV *p* = 0.79, laminin *p* = 0.30, fibronectin *p* = 0.44).

### Vascular sprout formation in HPL-based matrices induced by spherical MSC/HUVEC aggregates

**Fig 3:**
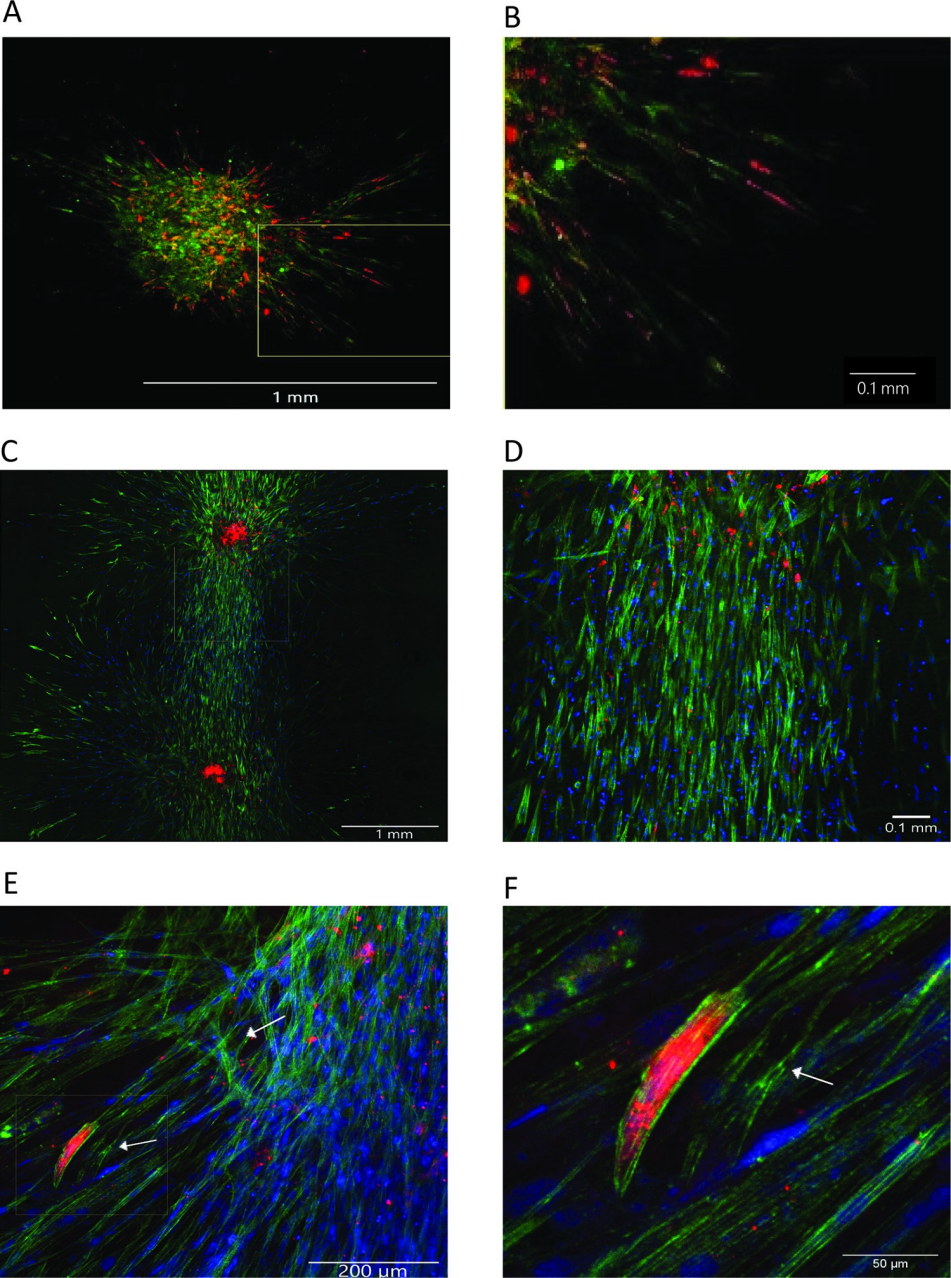
Spherical aggregates in HPL-based matrices. Spherical aggregates consisting of 4500 MSCs and 500 HUVECs were generated by the hanging drop technology and subsequently embedded in 20% HPL matrices. MSCs stained with CellTracker^TM^ Green and HUVECs stained with CellTracker^TM^ Red were co-cultured to generate spherical aggregates. These aggregates were embedded into HPL-based matrices for 6 days to see vascular sprouts emanating from the aggregates (A, B). A representative image of two aggregates embedded into the HPL-based matrices in approximately 3 mm showed interconnection of the sprouts after 21 days of cultivation (C, D). Actin filaments of MSCs and HUVECs were stained with phalloidin (green) and HUVECs were stained by the endothelial specific marker vWF (red) (C-F). Branch points and lumen formation are indicated by white arrows.

To study the supportive role of MSCs in *de novo* vessel formation by HUVECs, we used 20% HPL-based scaffolds. Before 3D spherical aggregate formation, MSCs were labeled with CellTracker^TM^ Green and HUVECs with CellTracker^TM^ Red. The aggregates were embedded in HPL-based matrices in distances of 2–5 mm. A digital documentation system was used to visualize vascular development over a period of 21 days (Fig 3). Spherical aggregates consisting only of HUVECs were instable and could not be transferred to the matrices, whereas MSCs alone formed stable spheroids (S4A Fig). For co-culture aggregates, the support of autologous MSCs was required to ensure viability of the HUVECs. HUVECs cultured by hanging drop with non-autologous MSCs showed a round morphology indicating dying cells (S4B Fig).

**Fig 4:**
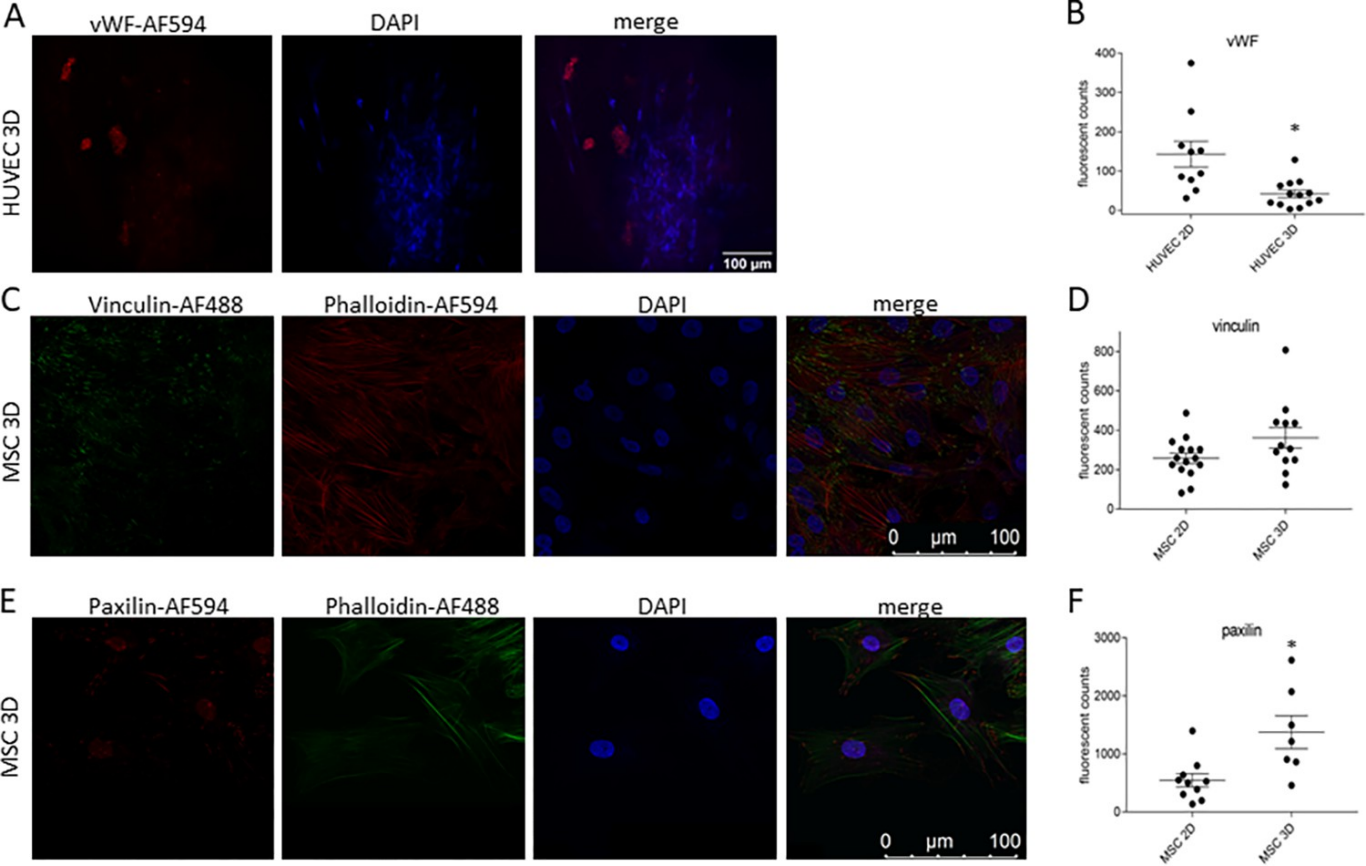
Different expression levels of the endothelial marker vWF and focal adhesion proteins in the 3D spheroids compared to 2D cultures. The expression of the endothelial marker vWF (A) and the focal adhesion proteins vinculin (C) and paxillin (E) are shown in 3D spheroids by confocal microscopy. The fluorescence intensity of vWF (B) in HUVECs, vinculin (D) and paxillin (F) in MSCs were measured based on confocal fluorescent images of 3D aggregates and 2D cell culture using ImageJ. For the analysis of the expression levels of vWF, and the focal adhesion proteins vinculin and paxillin in MSCs in 2D compared to 3D culture, a two-tailed *Student’s* t-test was performed using GraphPad Prism 7. * *p*<0.05.

We showed that the spatial organization of cellular outgrowths into the initially avascular translucent HPL-based matrices occurred within the first five days of cultivation (Fig 3A and 3B). These outgrowths changed their appearance to a spindle-shape cell morphology, forming cellular strands that continued to grow towards neighboring strands showing the same morphology (Fig 3C and 3D). These newly formed sprouts developed in different planes within the HPL-based matrices to form cord-like structures with a mix of labeled HUVECs and MSCs (Fig 3E and 3F). Within these newly formed sprouts, HUVECs showed nuclear elongation and occasionally formed tip cells positive for von Willebrand factor vWF (Fig 3E and 3F). Generation of vascular branch points and a lumen are important maturation steps of newly formed blood vessels during their establishment (Fig 3F). However, compared to 2D co-cultures, the HUVECs in our 3D spheroids expressed lower levels of vWF (Fig 4A and 4B, vWF *p* = 0.0034, n_2D_ = 10 n_3D_ = 13), indicating a decreased angiogenesis potency.

Analyzing the dynamics of the adherens junctions, we found that the cytoskeletal protein vinculin, which is associated with focal adhesion sites in the ECM, and the focal adhesion adaptor protein paxillin are higher expressed in the MSCs of spheroids compared to those in 2D cultures (Fig 4C–4F, vinculin *p* = 0,071 n_2D_ = 15 n_3D_ = 12, paxillin *p* = 0,008 n_2D_ = 10 n_3D_ = 7)

**Fig 5:**
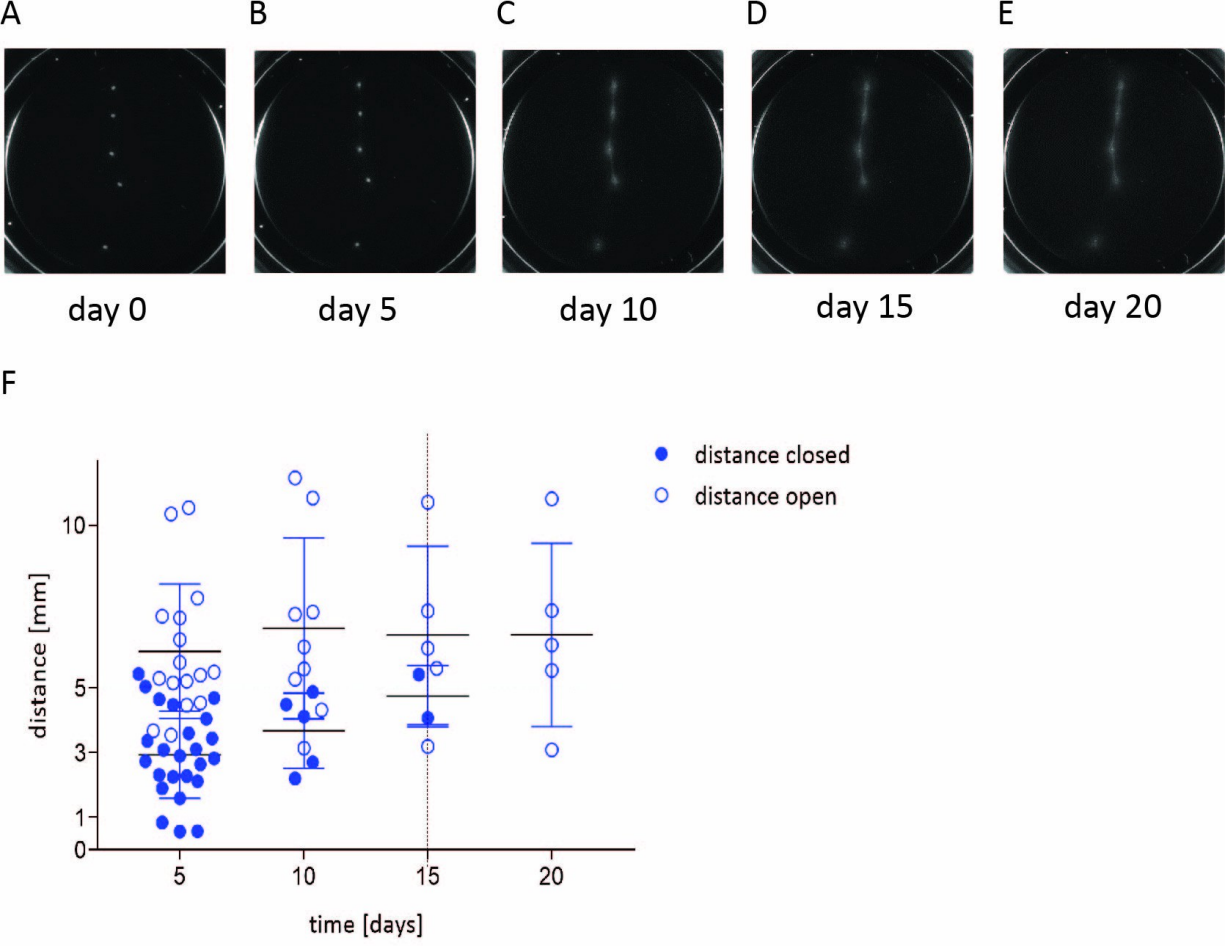
Vascular sprout formation in HPL-based matrices. The capability of two spherical aggregates consisting of 4500 MSCs and 500 HUVECs to bridge distances of up to 5 mm with their vascular sprouts was investigated using phase contrast microscopy (A-E). The closure time of two adjacent spherical aggregates was recorded over a period of 20 days. The joining of vascular sprouts from two adjacent spherical aggregates was recorded at the day indicated with successful joining (filled circles) and failure to close (open circles) (F).

Besides, we showed that the newly formed vascular structures showed branch points and generated a lumen in our static system in the total absence of a blood circulation introducing flow (Fig 5E).

No evidence was found for a mixed endothelial/mesenchymal phenotype or cross-differentiation of MSCs to endothelial cells in our experimental setting (S5 Fig). The endothelial marker vWF could not be detected in MSCs stained with CellTracker^TM^ Red after co-culturing with HUVECs for 21 days (S5 Fig). MSCs presumably have a scaffolding function in the 3D spheroids to ensure their stability and support the viability and proliferative function of HUVECs.

The vascular sprouts that emanated from a spherical aggregate consisting of co-cultured MSCs and HUVECs could bridge distances up to 5 mm to form an interconnected network with adjacent aggregates showing the same morphology after 10–15 days of cultivation (Fig 5).

### Binding of MSCs to adherent HUVEC layer under flow

The capability of MSCs to bind HUVECs under dynamic fluid conditions mimicking the blood flow in venous vessels using a microfluidic chamber was investigated by the BioFlux^®^200 device that enabled multiple temperature-controlled flow assays to run in parallel. In contrast to commercial HUVECs, which are usually in passage 4–5, we used cells of passage 1. Previously, we could show that cells of higher passages showed less VE-cadherin-positive junctions and respond differently in their orientation to flow in the BioFlux^®^200 compared to HUVECs in passage 1. MSCs of passages >3 showed an altered mitochondrial morphology and the formation of stress fibers [21].

**Fig 6:**
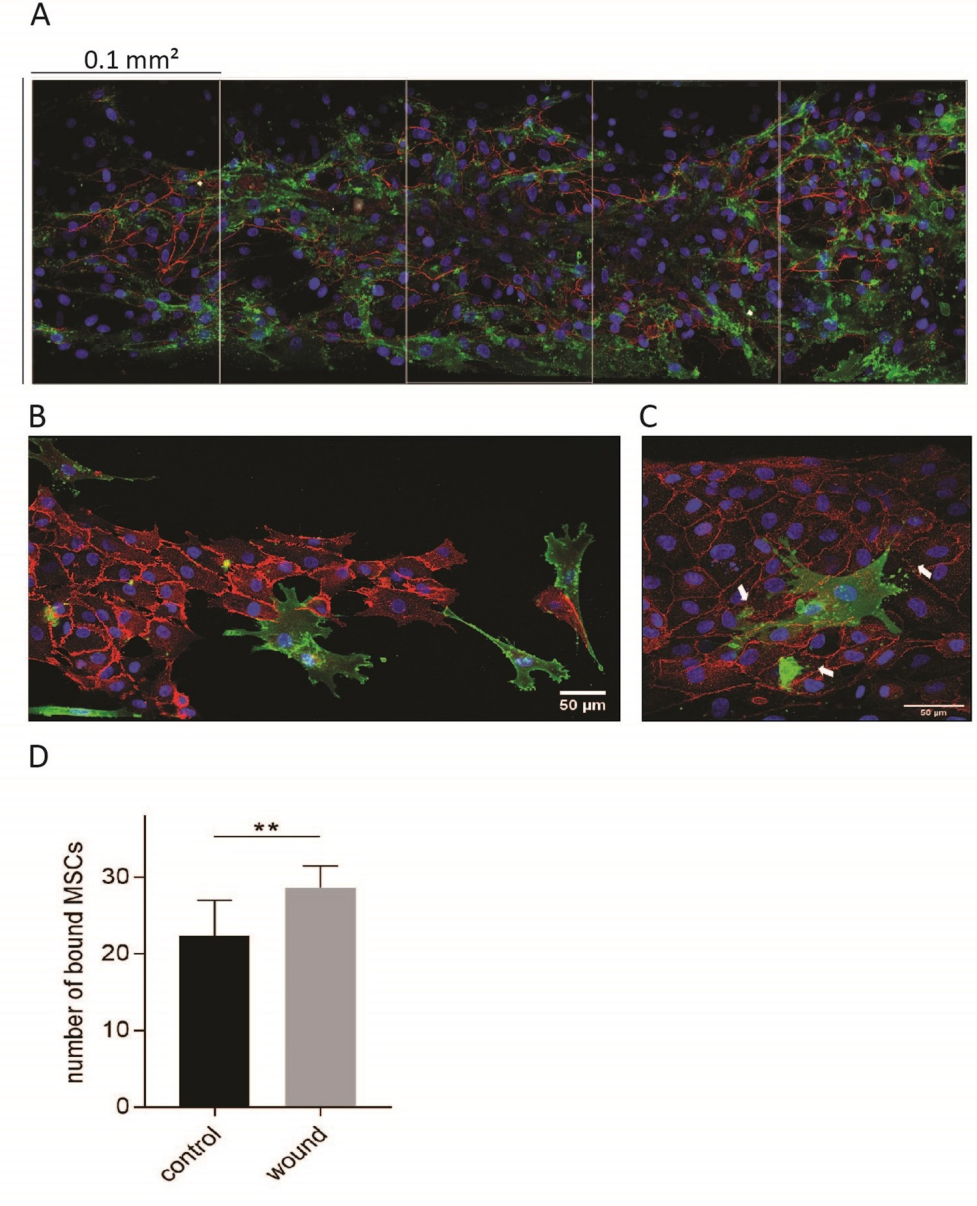
MSCs binding to a layer of HUVECs under flow in a microfluidic system. MSCs in fluid phase were applied under 2 dyne/mm^2^ shear rate to bind to a layer of HUVECs in a BioFlux^®^200 system with a total chamber area is 0.525 mm^2^ (1500 mm x 350 mm) and one sector indicating 0.1 mm^2^ (A). A representative area of damaged HUVECs stained with CD144 (VE-cadherin, red) and bound MSCs stained with CD90 (green) (B, C). Some MSCs formed nano-tubular extrusions to interact with the HUVECs (red) (D). Statistical analysis was performed by an unpaired, two-tailed t-test (control n = 34, wound n = 6) using GraphPad Prism 7.

MSCs applied in the fluid phase bound HUVECs in a density of 35–40 MSCs/0.1 mm^2^ when a flow rate of 2 dyne/cm^2^ was applied (Fig 6A). The shear rate of 2 dyne/cm^2^ represents the physiological value in human venules with diameters ranging from 20–70 μm [25, 26]. In case of cell damage induced by laser radiation to the HUVECs, MSCs bound the HUVECs with a higher frequency (Fig 6D, *p =* 0.0027, control n = 34, wound n = 6). Some MSCs formed long nano-tubular extrusions (Fig 6B, arrow), others directly bound to the damaged HUVECs.

## Discussion

Tissue regeneration relies on neo-vessel formation, a process that requires MSCs to support ECs during initiation and development of vascular sprouts. Tissue damage with vascular injury leads to leakage of plasma components into the damaged site and the generation of an early provisional matrix consisting of fibrin-rich polymers with interspersed cross-linked fibronectin and platelets [27–30]. The clotting response and the release of platelet α-granule content into the fibrin-rich provisional matrix stop bleeding and create a basic scaffold for MSC-EC interaction [27]. Next to fibrinogen, more than 300 bioactive substances are released by activated platelets that can potentially interfere with MSC-EC communication [31]. We argued that fibrin-based matrices that incorporate the platelet secretome serve as ideal scaffolds to create new vessels close to physiological conditions [27–30]. Here, we established an HPL-based matrix to investigate new vessel formation induced by HUVECs which requires the support of MSCs. HUVECs co-cultured with autologous MSCs generated stable spherical aggregates, an ideal tool for vascular engineering, since MSCs provide the ECM components necessary for aggregate stability, such as collagen type I, fibronectin, laminin, and collagen type IV [16, 17, 32]. This is of importance because the early provisional fibrin-rich matrix is replaced during the process of healing by locally produced fibronectin, laminin, and proteoglycans, generated by invading cells of the mesenchymal lineage [27, 33]. We demonstrated that MSCs within spherical aggregates provide collagen type I and IV as well as fibronectin and laminin important for aggregate stability (Fig 2), while HUVECs did not add to ECM formation under these conditions. The fibronectin assembly is driven by a complex process of cell binding, molecular extension of the protein through physical forces to expose multiple cryptic self-association sites [28, 29]. In the polymerized state, fibronectin can be considered as a scaffold signaling protein favoring new vessel formation [29, 30, 34–38]. Additionally, the MSCs within the 3D spheroids expressed higher levels of focal adhesion-associated proteins important for cell-cell contact and adhesion to the ECM (Fig 4C–4F). By inducing junctional remodeling in the 3D culture, MSCs may maintain vascular integrity during sprouting.

In this study we verified the formation of vascular sprouts originating from the inserted spherical MSC-HUVEC aggregates into the primarily avascular HPL-based matrices. When spherical MSC-HUVEC aggregates were embedded into HPL gels at distances of up to 5 mm between the aggregates, the sprouts grew towards the adjacent aggregate showing the same morphology and joined to a connected network. Interestingly, these outgrowths also formed a lumen, similar to results previously shown by *Ruger et al* [39].

Further, we found that MSCs bind to autologous HUVECs under dynamic conditions mimicking the blood flow in blood vessels [40]. The shear rate of 2 dyne/cm^2^ represents the physiological values in human venules with diameters ranging from 20–70 μm [25]. When the perfused HUVEC layer was damaged by UV laser beam irradiation, MSCs bound preferentially to the HUVECs located at the rim of damage. To contact the damaged HUVECs, MSC generated long nano-tubular extrusions (Fig 6B and 6C).

## Conclusion

Here, we provide evidence that spherical MSC/HUVEC aggregates can initiate new vessel formation and form vascular sprouts by self-induction in HPL-based matrices. These newly formed sprouts can bridge distances of up to 5 mm to join and form an interconnected network with an adjacent aggregate having the same morphology. Although we found no evidence for trans-differentiation of MSCs into ECs, characterized by vWF expression, in our 3D system [36], we could show that MSCs support ECs in the initiation process of new vessel formation *in vitro*. Our results give evidence for the capability of MSCs to interact actively with HUVECs in the process of vessel formation in artificial HPL-based matrices without the requirement of additional pro-vascular bioactive molecules. From the clinical view, these assays can give further information on the ability of MSCs to contribute to the regeneration of vascular defects.

## Supporting information

S1 Fig Phenotypic characterization of MSCs. MSCs were phenotypically characterized by flow cytometry using the stem cell markers CD73, CD90 and CD105.
(TIF)

S2 Fig Extracellular matrix formation by MSCs in spherical aggregate cultivated in MSC-GM. Spherical MSC aggregates were cultivated for the time indicated (3d, 6d, 9d and 12d) and cryo-sectioned and stained with mAbs specific form collagen type I (A-C), collagen type IV (D-G), laminin (H-K) and fibronectin (I-L) (red) and counterstained with CD90 (green). For nuclei staining DAPI was applied (blue).
(TIF)

S3 Fig Extracellular matrix formation by MSCs in spherical aggregate cultivated in MSC-BM with 8% HPL. Spherical MSC aggregates were cultivated for the indicated time (3d, 6d, 9d and 12d), cryo-sectioned and stained with mAbs specific form collagen type I (A-C), collagen type IV (D-G), laminin (H-K) and fibronectin (I-L) (red) and counterstained with CD90 (green). For nuclei staining DAPI was applied (blue).
(TIF)

S4 Fig Generation of stable spheroids using autologous MSCs and HUVECs. A) Spherical aggregates consisting of HUVECs only, MSCs only, and autologous MSCs and HUVECs in a ratio of 9:1 in hanging drops observed under a light microscope. Spheroids that contain only HUVECs are instable, whereas the addition of autologous MSCs leads to stable spheroid formation. B) Non-autologous (top) and autologous (bottom) MSCs stained with CellTracker^TM^ Green and HUVECs stained with CellTracker^TM^ Red were cultured by hanging drop. Using non-autologous MSCs, the HUVECs showed a round morphology indicating dying cell. In the autologous spheroids the HUVECs are viable, mostly located in the sprouts.
(TIF)

S5 Fig Determination of cross-differentiation of MSCs into an endothelial phenotype. MSCs were stained with CellTracker^TM^ Red and co-cultured with HUVECs in HPL-based medium for 21 days. As controls, stained MSCs and unstained HUVECs, respectively, were cultured in HPL-based medium for 21 days. After fixation, cells were stained with a rbAb specific for vWF as endothelial marker, counterstained with anti-rabbit AF488 (green), and DAPI to highlight the nuclei (blue). After 21 days of co-culture with HUVECs in HPL-based medium, no expression of vWF was detected in MSCs.
(TIF)
