# Supplementary figures and images for: Mesenchymal stem cells support human vascular endothelial cells to form vascular sprouts in human platelet lysate-based matrices
Source: PLoS One. 2022 Dec 15;17(12):e0278895. doi: 10.1371/journal.pone.0278895 (PMC9754269; doi:10.1371/journal.pone.0278895)

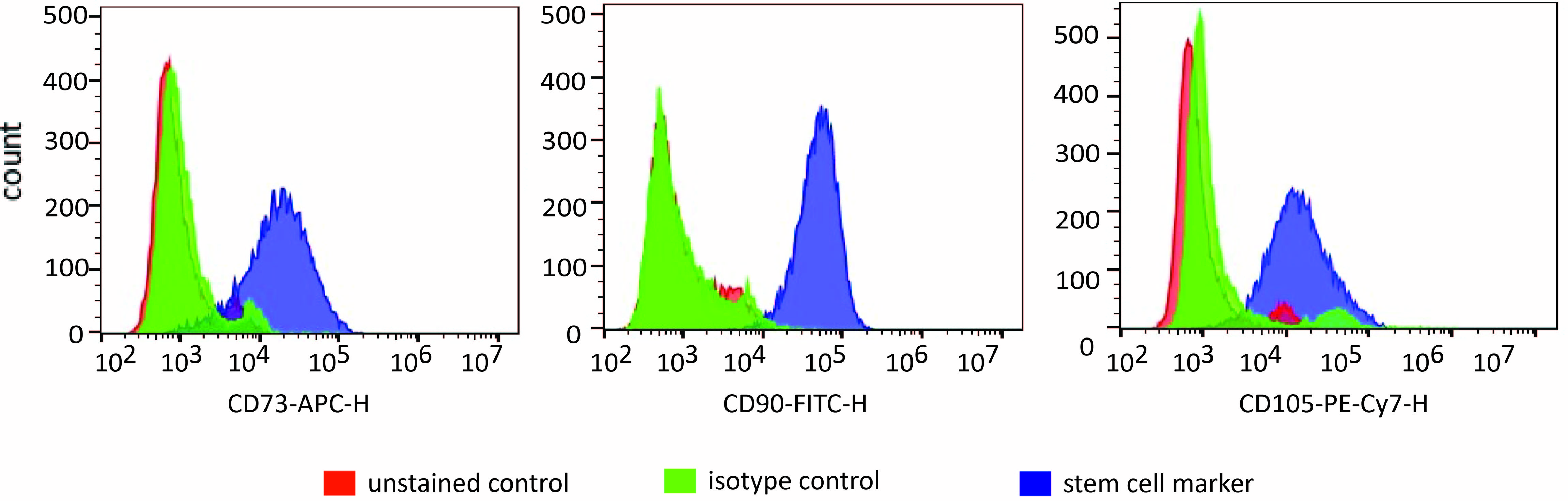

Supplement: S1 Fig — MSCs were phenotypically characterized by flow cytometry using the stem cell markers CD73, CD90 and CD105. (TIF) [file pone.0278895.s001.tif]

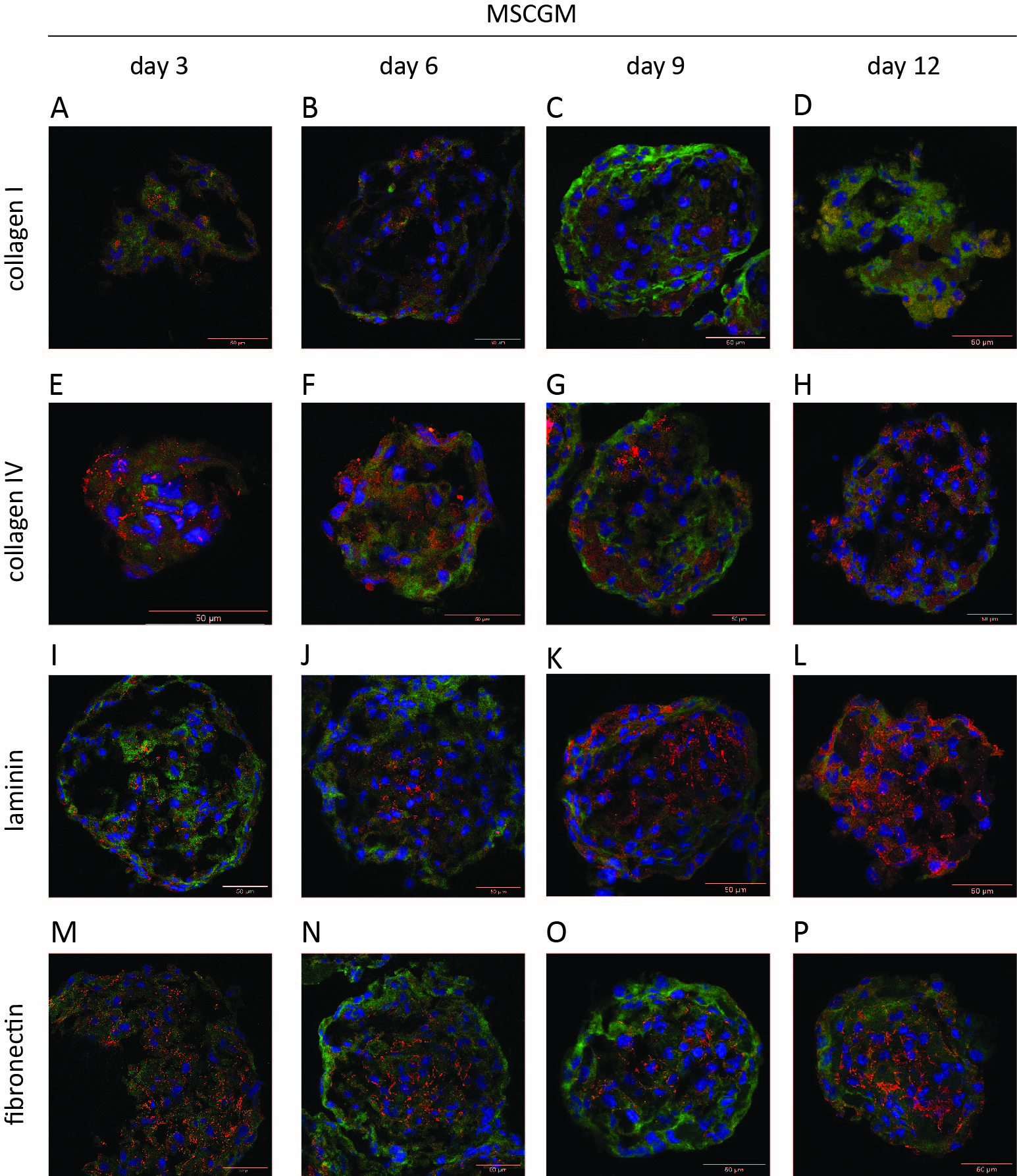

Supplement: S2 Fig — Spherical MSC aggregates were cultivated for the time indicated (3d, 6d, 9d and 12d) and cryo-sectioned and stained with mAbs specific form collagen type I (A-C), collagen type IV (D-G), laminin (H-K) and fibronectin (I-L) (red) and counterstained with CD90 (green). For nuclei staining DAPI was applied (blue). (TIF) [file pone.0278895.s002.tif]

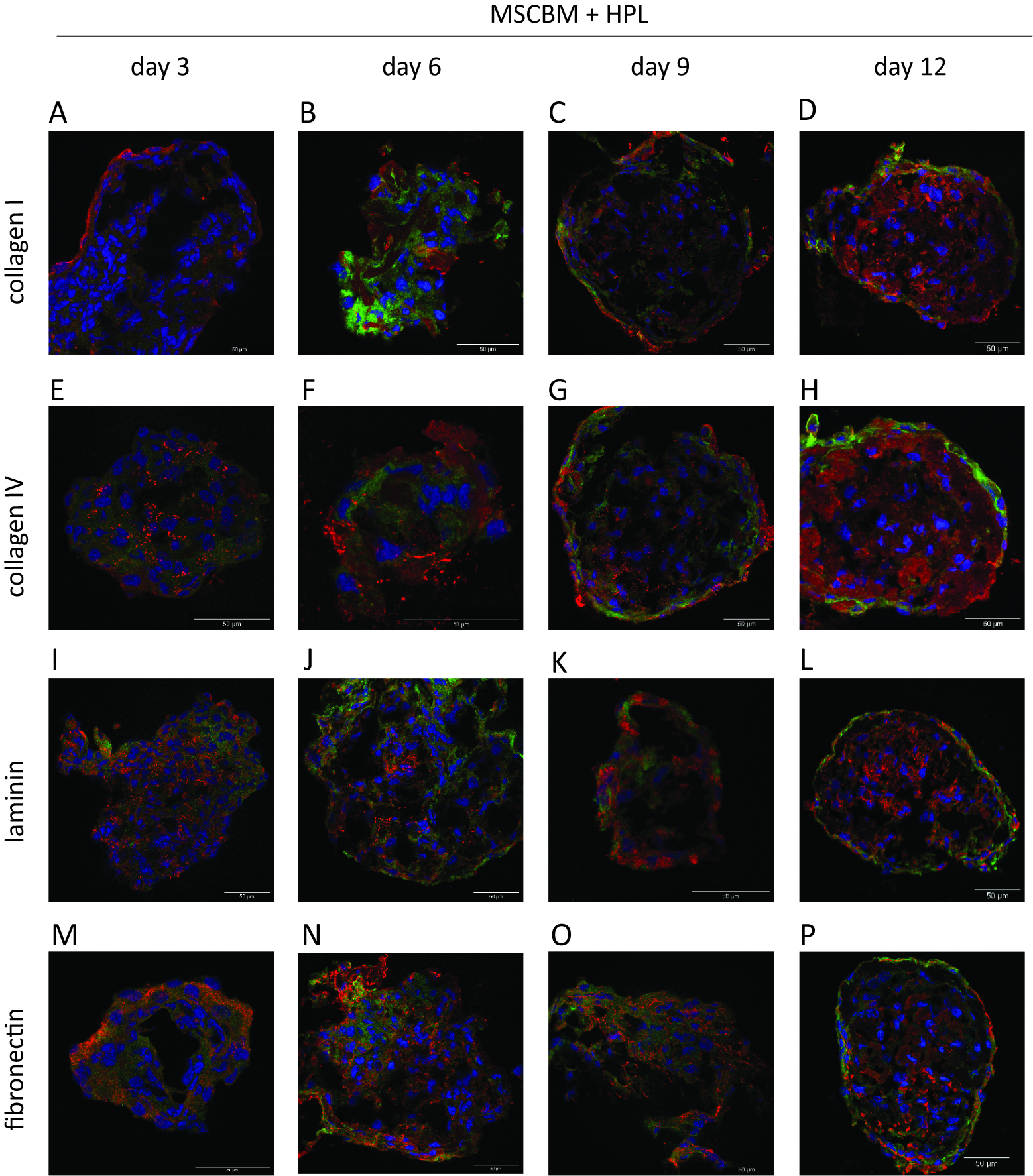

Supplement: S3 Fig — Spherical MSC aggregates were cultivated for the indicated time (3d, 6d, 9d and 12d), cryo-sectioned and stained with mAbs specific form collagen type I (A-C), collagen type IV (D-G), laminin (H-K) and fibronectin (I-L) (red) and counterstained with CD90 (green). For nuclei staining DAPI was applied (blue). (TIF) [file pone.0278895.s003.tif]

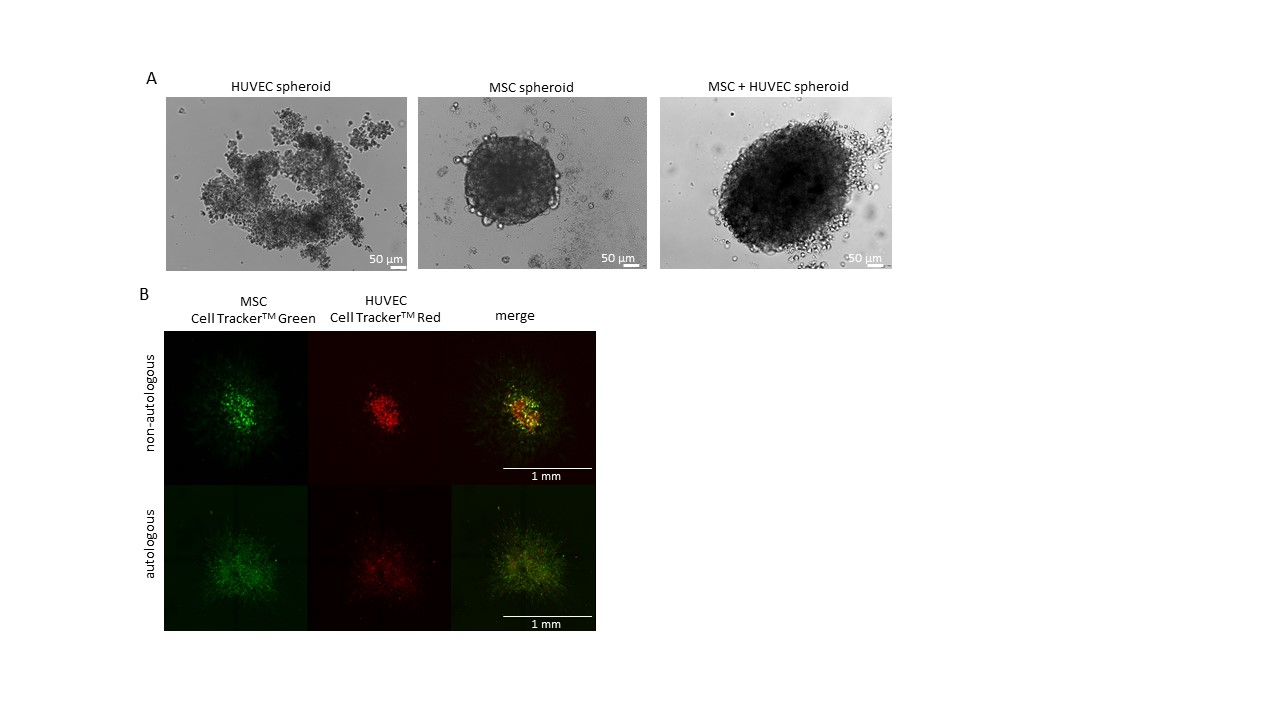

Supplement: S4 Fig — A) Spherical aggregates consisting of HUVECs only, MSCs only, and autologous MSCs and HUVECs in a ratio of 9:1 in hanging drops observed under a light microscope. Spheroids that contain only HUVECs are instable, whereas the addition of autologous MSCs leads to stable spheroid formation. B) Non-autologous (top) and autologous (bottom) MSCs stained with CellTrackerTM Green and HUVECs stained with CellTrackerTM Red were cultured by hanging drop. Using non-autologous MSCs, the HUVECs showed a round morphology indicating dying cell. In the autologous spheroids the HUVECs are viable, mostly located in the sprouts. (TIF) [file pone.0278895.s004.tif]

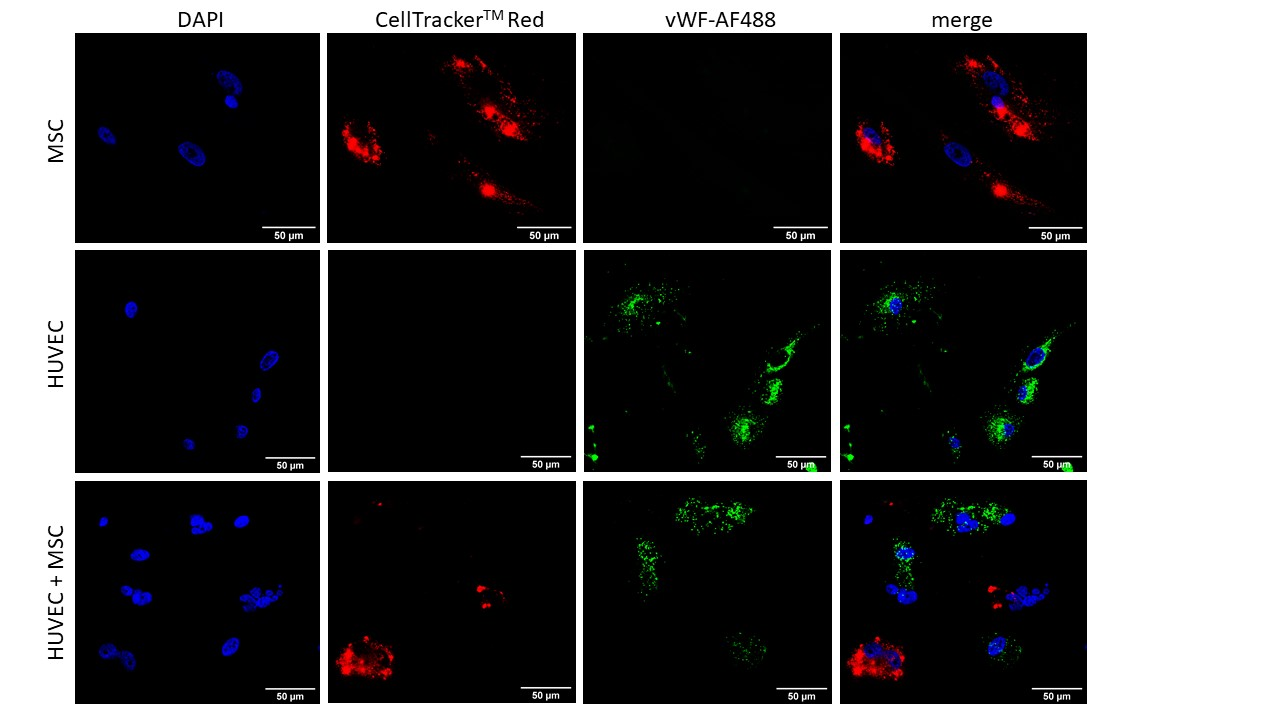

Supplement: S5 Fig — MSCs were stained with CellTrackerTM Red and co-cultured with HUVECs in HPL-based medium for 21 days. As controls, stained MSCs and unstained HUVECs, respectively, were cultured in HPL-based medium for 21 days. After fixation, cells were stained with a rbAb specific for vWF as endothelial marker, counterstained with anti-rabbit AF488 (green), and DAPI to highlight the nuclei (blue). After 21 days of co-culture with HUVECs in HPL-based medium, no expression of vWF was detected in MSCs. (TIF) [file pone.0278895.s005.tif]
